# Differentiating the impact of ambient temperature on hospitalization due to cause-specific pneumonias: an individual-level, case-crossover study

**DOI:** 10.3389/fpubh.2025.1615337

**Published:** 2025-10-20

**Authors:** Xiaozhen Su, Lu Zhou, Yingmin Tao, Renjie Chen, Juan Xie

**Affiliations:** ^1^Department of Environmental Health, School of Public Health, Key Lab of Public Health Safety of the Ministry of Education, Fudan University, Shanghai, China; ^2^Division of General Practice, The Fifth People's Hospital of Shanghai, Fudan University, Shanghai, China; ^3^Center of Community-Based Health Research, Fudan University, Shanghai, China

**Keywords:** pneumonia, hospital admission, ambient temperature, climate change, case-crossover study

## Abstract

**Introduction:**

Associations between exposure to ambient temperature and the risks of hospital admission for overall or a specific type of pneumonia were reported in prior studies. However, few studies have systematically explored the potentially differential impacts of ambient temperature on hospitalization for cause-specific pneumonias.

**Methods:**

We performed a time-stratified, case-crossover study based on individual-level pneumonia-related hospital data with pathogen identification results in Shanghai, the largest metropolis in China from 2013 to 2019. Conditional logistic regression combined with distributed lag non-linear model was used to estimate effects of extreme temperature.

**Results:**

We included a total of 6,277 hospitalized cases of pneumonias with various causes. The observed exposure-response curve for the association between temperature and pneumonias was nonlinear, with significantly elevated risks in high temperature. The hot-related risk appeared on lag 0–1 day, and became non-significant on lag 5–10 day, with differential lag patterns in various subtypes of pneumonias. Extreme high temperature exhibited the largest effect estimate in hospitalization from infectious pneumonia [relative risk: 2.55, 95% confidence interval (CI): 1.58, 4.13], followed by bacterial pneumonia (2.16, 95% CI, 1.15, 4.04), and total pneumonia (1.96, 95% CI, 1.40, 2.74). Stronger relationships were observed in females in stratified analyses. The effect estimates remain robust after adjusting for air pollutants and various model parameters.

**Discussion:**

As pneumonias cause a huge disease burden worldwide, our study adds heat-related risk assessments on hospitalization due to cause-specific pneumonias, which is beneficial for prevention and management of patients with specific subtypes of pneumonia in urban areas.

## 1 Introduction

Pneumonia is a common and life-threatening respiratory disease with the characteristic of recurrent attacks and a huge burden on economy and healthcare. The Global Burden of Disease (GBD) study estimated that lower respiratory tract infections (LRIs) were the fourth top cause of death globally, causing 2.49 million deaths worldwide in 2019 (1). In addition, the morbidity of pneumonia in China is 7.13 per 1,000 person-years (2), with hospitalization rate for pneumonia increasing at an annual rate of 15.5% and a 30-day mortality rate of 2.4% (3), which leaded to a loss of labor productivity and a huge economic burden on the healthcare system. The causes of non-infectious pneumonia include pollen, dust and bird feathers, while the causes of infectious pneumonia include bacteria, viruses, fungi and mycoplasma (4). The epidemic season, susceptible population and clinical treatment may vary by the cause of pneumonia. Therefore, exploring the modifiable risk factors of cause-specific pneumonias and identifying the vulnerable population is important for comprehensive and nuanced disease management.

GBD 2019 identified non-optimal ambient temperature as a new environmental risk factor, which warrants urgent attention in disease prevention under a changing climate (5–7). Within the framework of global warming, the frequent and widespread heat events can lead to elevated risks of diverse adverse health consequences, including pneumonia. Numerous epidemiological evidence linked non-optimum temperature with elevated risks of pneumonia morbidity and mortality, but previous findings are still inconsistent (8–11). For instance, a number of studies in America (12), South Korean (13), China (14) and South Africa (15), reported that short-term exposure to ambient low temperature could significantly elevate the mortality and morbidity risk of pneumonia and last for 2–3 weeks, but a study in America found no significant association between extreme cold and pneumonia mortality (16). Several studies demonstrated that extreme high temperature was associated with elevated risk of pneumonia (17, 18), but a few studies suggested a non-significant heat-pneumonia association (19–21). Moreover, the majority of prior investigations utilized ecological time-series design based on aggregated daily data of pneumonias, which may lead to difficulty in controlling for individual confounding effects and causal inference. In addition, previous studies assessed exposure level of ambient temperature by fixed-site weather stations, whose relatively far distance may lead to larger exposure errors than high-resolution exposure data.

Notably, previous studies mainly assessed the effects of non-optimum temperature on total pneumonia or a specific subtype of pneumonia (2, 22, 23). For instance, a time-series investigation in Beijing, China, found that the relative risk (RR) for extreme cold temperature was 2.12 for pneumonia hospital visits, whereas the effect of extreme high temperature was non-significant (20). Another time-series study in Fukuoka, Japan, reported that the hospitalization risk of mycoplasma pneumonia increased 16.9% for each 1 °C increase in temperature (24). The systematic exploration for the potentially differential impacts of non-optimum temperature exposure on cause-specific pneumonias is limited and the results remain inconsistent (25, 26).

Therefore, we performed an individual-level, time-stratified, case-crossover study to evaluate effects of short-term exposure to non-optimum ambient temperature on hospitalizations from cause-specific pneumonias in Shanghai, China.

## 2 Methods

### 2.1 Study population

Shanghai is the largest metropolitan area in China, and experiences a subtropical monsoon-influenced maritime climate due to its location on the western coast of the Pacific Ocean, characterized by pronounced seasonal changes, elevated humidity levels, and significant monsoon activity. Hot and humid summers are typically accompanied by heatwaves, while cold and moist winters feature periodic cold spells with the absence of centralized heating. These climatic factors, together with high population density and an increasingly aging demographic in this highly urbanized city, heighten its susceptibility to extreme temperature events.

From January 1^st^, 2013 to December 31^st^, 2019, we collected the individual inpatient records from the hospital information system from the Shanghai Fifth People’s Hospital Affiliated to Fudan University, a comprehensive public medical institution located in Shanghai, China. We only included the patients who had a diagnosis of pneumonias (B35–B49, J12–J18, J60–J70) with pathogen identification results sorted by the International Classification of Diseases, 10th Revision (ICD-10), and excluded patients with residence outside of Shanghai. The medical records included pathogen identification results, as well as basic demographic characteristics such as age, sex, residential address and date of hospital admission. Pathogen detection and identification were performed using sputum specimens of hospitalized pneumonia patients. Bacterial and fungal detection was performed using sputum culture techniques, viral detection was carried out using polymerase chain reaction (PCR) technology, and mycoplasma detection was conducted using antigen–antibody techniques. We excluded individuals who had unclear basic information, smoking history and occupational exposure history to smoke or dust from our study. According to the pathogen detection and identification results, we assessed the following subtypes of pneumonia: non-infectious pneumonia and infectious pneumonia, which included bacterial, viral, fungal, and mycoplasma pneumonia.

### 2.2 Exposure assessment

For each pneumonia patient, we evaluated individual-level ambient temperature by matching the nearest gridded cell of exposure data to residential address of the patient. We obtained meteorological data from the fifth generation of European Reanalysis (ERA5) database, including daily mean temperature at 0.1° × 0.1° spatial resolution and relative humidity at 0.25° × 0.25° spatial resolution (27). We obtained data of six criteria air pollutants to account for the confounding effects, including fine particulate matter (PM_2.5_), coarse particulate matter (PM_2.5–10_), nitrogen dioxide (NO_2_), ozone (O_3_, maximum 8-h average), carbon monoxide (CO) and sulfur dioxide (SO_2_). The daily average concentrations of PM_2.5_, PM_2.5–10_, NO_2_, and O_3_ were collected from established satellite model data at a 1-km spatial resolution, which had been demonstrated to have high accuracy in urban areas and more comprehensive prediction in suburban areas (28, 29). We obtained the daily concentrations of CO and SO_2_ from the National Urban Air Quality Real-time Publishing Platform, nearest to the residential address. Moreover, data of precipitation, wind speed, and sunshine duration were derived from the National Urban Air Quality Real-time Publishing Platform^1^.

### 2.3 Study design

We used an individual-level, time-stratified, case-crossover study design to estimate the impact of extreme high temperature on hospitalizations from cause-specific pneumonias from January 1st, 2013 to December 31st, 2019 in Shanghai, China. This self-matching study design is commonly utilized in recent environmental epidemiological studies (30, 31), which can automatically control the time trends and relatively stable factors over a brief period, such as sex, age, chronic diseases, economic status, and lifestyle. We defined the day of hospitalization for pneumonia as the case day, and the same day of the week, month, and year as the control days for each case. For instance, if a pneumonia patient was admitted to hospital on Friday, April 17th, 2015, then this day would be defined as the case day and the remaining Fridays in April 2015 (i.e., April 3rd, 10th, and 24th) as the control days.

### 2.4 Statistical analyses

To estimate effects and nonlinear exposure-response relationship of non-optimum temperature on hospitalizations from pneumonias of various causes, we utilized conditional logistic regression models combined with the distributed lag nonlinear model (DLNM). The DLNM model captures the exposure-response association and temporal lag effect of temperature on health events by constructing a cross-basis that incorporates both the exposure-response and lag dimensions, which effectively controls for collinearity between lag days while maintaining modeling flexibility. We first established a single-variable model to assess the impact of non-optimum temperature on various types of pneumonias. Specifically, we *a priori* used a natural cubic spline with three internal knots at equidistant percentiles of temperature distribution (the 25th, 50th, and 75th) to build a cross-basis function of temperature. We selected 21 days as the maximum lag and a natural cubic spline with 3 degrees of freedom (*df*) to depict lag pattern of extreme low and high temperature because previous findings showed that acute effects of ambient temperatures on pneumonias could last for about 3 weeks (30–32). In addition, we accounted for various weather variables [relative humidity with a natural cubic spline with 3 *df*, precipitation, wind speed, and sunshine duration on the current day of exposure], and public holidays in the model.

We firstly defined the reference temperature as the minimum morbidity temperature and the extreme low and high temperature as the 1st and 99th percentiles of the temperature distribution according to prior findings (26). We then plotted lag patterns in impacts of extreme non-optimum temperature on cause-specific pneumonias and achieved the relative risks and their 95% confidence intervals (95% CIs) of extreme low and high temperature compared to the reference temperature over the period of significant effect in the main analyses. Moreover, the range of temperature distribution to depict exposure-response relationship curves between extreme temperature and cause-specific pneumonias was from the 1st percentile to the 99th percentile, which aimed to reduce statistical uncertainty caused by the limited sample size at extreme temperatures.

In addition, we conducted subgroup analyses by sex (male and female) and age (<60 years old and ≥60 years old) to identify vulnerable subgroups. The *Z* test was used to test the statistical differences between stratum-specific effects. Furthermore, six criteria atmospheric pollutants were included one by one, and all six air pollutants were adjusted at once in the main model to test our findings’ robustness. We also change various model parameters to test the robustness, including the lag period of relative humidity, the spline function and *df* of relative humidity, the basic functions and *df* of DLNM model. Moreover, the generalized variance inflation factor (GVIF) was used to assess the potential autocorrelation and collinearity in our models.

R version 4.2.1 with *“dlnm”* and *“survival”* packages was used to conduct all analyses.

## 3 Results

### 3.1 Descriptive statistics

From January 1st, 2013, to December 31st, 2019, a total of 6,277 cases hospitalized for pneumonia were included in the analyses, including 2,928 (46.6%) cases for non-infectious pneumonia and 3,349 (53.4%) for infectious pneumonia. Among the infectious pneumonias, 1,793 (28.6%) were bacterial pneumonia infections, 533 (8.5%) were viral pneumonia infections, 797 (12.7%) were fungal pneumonia infections, 1,003 (16.0%) were mycoplasma pneumonia infections and 33 (0.5%) were other pneumonia infections. Of all patients, 50.9% were female and 62.8% were aged above 60 years old (Table 1).

**Table 1:** Summary statistics of cases hospitalized for cause-specific pneumonias in Shanghai, China, from 2013 to 2019.

Variable	Total	Age < 60	Age ≥ 60	Male	Female
Total pneumonia, *n* (%)	6,277 (100)	2,333 (37.2)	3,944 (62.8)	3,084 (49.1)	3,193 (50.9)
Non-infectious pneumonia, *n* (%)	2,928 (46.6)	1,037 (35.4)	1891 (64.6)	1,466 (50.1)	1,462 (49.9)
Infectious pneumonia, *n* (%)	3,349 (53.4)	1,296 (38.7)	2053 (61.3)	1,618 (48.3)	1731 (51.7)
Bacterial pneumonia, *n* (%)	1793 (28.6)	691 (38.5)	1,102 (61.5)	911 (50.8)	882 (49.2)
Viral pneumonia, *n* (%)	533 (8.5)	245 (46.0)	288 (54.0)	222 (41.7)	311 (58.3)
Fungal pneumonia, *n* (%)	797 (12.7)	108 (13.6)	689 (86.4)	384 (48.2)	413 (51.8)
Mycoplasma pneumonia, *n* (%)	1,003 (16.0)	604 (60.2)	399 (39.8)	456 (45.5)	547 (54.5)
Other pneumonias, *n* (%)	33 (0.5)	13 (39.4)	20 (60.6)	15 (45.5)	18 (54.5)

As shown in Table 2, the average daily mean temperature and relative humidity were 16.4 °C [standard deviation (SD) = 8.7] and 73.1% (SD = 14.3), respectively. Daily average levels of other weather variables were 156.5 mm (SD = 689.6) for precipitation, 2.6 m/s (SD = 1.0) for wind speed, and 4.7 h (SD = 4.1) for sunshine duration. The average daily mean concentrations of atmospheric pollutants were 43.9 μg/m^3^ (SD = 27.5) for PM_2.5_, 28.1 μg/m^3^ (SD = 13.8) for PM_2.5–10_, 20.6 μg/m^3^ (SD = 8.2) for NO_2_, 84.8 μg/m^3^ (SD = 37.6) for O_3_, 16.9 μg/m^3^ (SD = 24.0) for SO_2_, and 3.1 mg/m^3^ (SD = 8.5) for CO. Weak-to-moderate correlations were detected between meteorological factors and atmospheric pollutants, with Spearman rank correlation coefficient ranging from 0.04 to 0.67 (Supplementary Table S1).

**Table 2:** Descriptive statistics on weather conditions and ambient air pollutants during the study period (2013–2019).

Variable	Mean	SD	P_25_	P_50_	P_75_	IQR
Temperature, °C	16.4	8.7	8.7	16.6	23.9	15.2
Relative humidity, %	73.1	14.3	64.7	75.9	83.6	18.9
Precipitation, mm	156.5	689.6	0.0	0.0	3.2	3.2
Wind speed, m/s	2.6	1.0	1.9	2.5	3.1	1.2
Sunshine duration, h	4.7	4.1	0.0	4.7	8.5	8.5
PM_2.5_, μg/m^3^	43.9	27.5	24.6	36.9	55.6	31.0
PM_2.5–10_, μg/m^3^	28.1	13.8	19.0	25.2	34.2	15.2
NO_2_, μg/m^3^	20.6	8.2	14.9	19.2	24.9	10.0
O_3_, μg/m^3^	84.8	37.6	56.4	78.4	108.6	52.2
SO_2_, μg/m^3^	16.9	24.0	7.1	10.3	16.2	9.1
CO, mg/m^3^	3.1	8.5	0.6	0.8	1.0	0.4

PM_2.5_, fine particulate matter; PM_2.5–10_, coarse particulate matter; NO_2_, nitrogen dioxide; O_3_, ozone; SO_2_, sulfur dioxide; CO, carbon monoxide; SD, standard deviation; P_25_, 25th percentile; P_50_, 50th percentile; P_75_, 75th percentile; IQR, interquartile range.

### 3.2 Regression results

**Figure 1:**
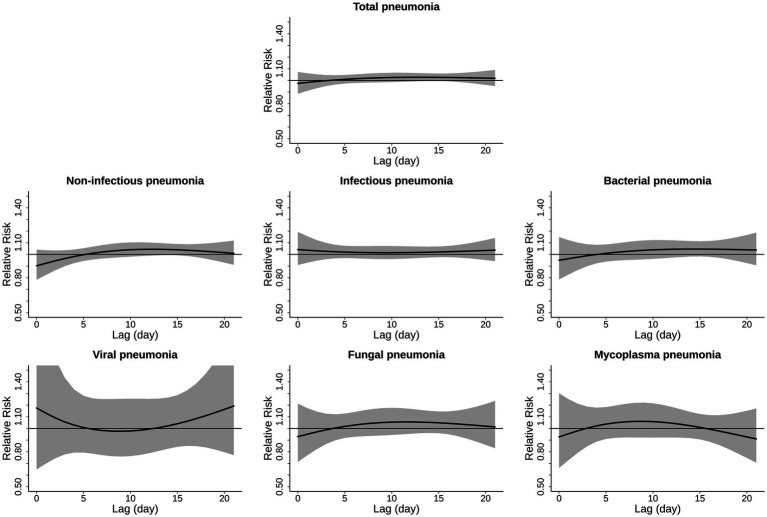
Lag-response curve for the associations of ambient low temperature with hospitalizations from cause-specific pneumonias. The associations were shown as relative risks and 95% confidence intervals of hospitalization at ambient low temperature (the 1st percentile of temperature distribution) compared to the corresponding reference temperatures on different lag days. The mean risk estimates and their 95% confidence intervals are shown by the black solid lines and the gray areas.

**Figure 2:**
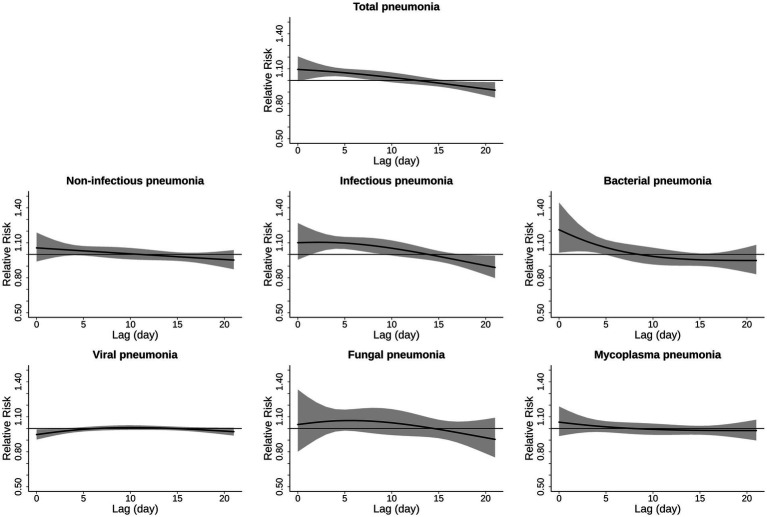
Lag-response curve for the associations of ambient high temperature with hospitalizations from cause-specific pneumonias. The associations were shown as relative risks and 95% confidence intervals of hospitalization at ambient high temperature (the 99th percentile) compared to the corresponding reference temperatures on different lag days. The mean risk estimates and their 95% confidence intervals are shown by the black solid lines and the gray areas.

Figures 1, 2 display the lag structures of associations between extreme low and high temperature and hospitalizations from pneumonias of various causes, respectively. These associations were shown as relative risks and 95% CIs of hospitalization at ambient low temperature (the 1st percentile of temperature distribution: 0.3, 0.2, 0.5, 0.3, 0.2, 0.3, and 0.8 °C, respectively) compared to the corresponding reference temperatures (the minimum morbidity temperatures: 20.3, 23.2, 19.5, 21.3, 26.0, 21.3, and 26.8 °C, respectively) on different lag days. The mean risk estimates and their 95% CIs are shown by the black solid lines and the gray areas. We did not observe significant effects of extreme low temperature on various pneumonias in our study (Supplementary Table S2). From Figure 1, we did not observe significant associations between extreme low temperatures and hospitalization risks of various pneumonias. Notably, our results of Figure 2 demonstrated that ambient high temperature could significantly increase the hospitalized risk of total, infectious, and bacterial pneumonias. The risks of extreme high temperature on total and infectious pneumonias occurred on lag 1 day, increased thereafter, peaked on lag 4 day, then decreased and became non-significant on lag 8–10 day. Heat effect of bacterial pneumonias was significant from lag 0 day to lag 5 day, which was different from other subtypes. Non-significant associations between ambient high temperature and non-infectious, viral, fungal and mycoplasma pneumonia were observed. Based on the lag patterns, we selected lag 0–10 days as the lag to derive risks of ambient high temperature and draw the exposure-response curves.

**Figure 3:**
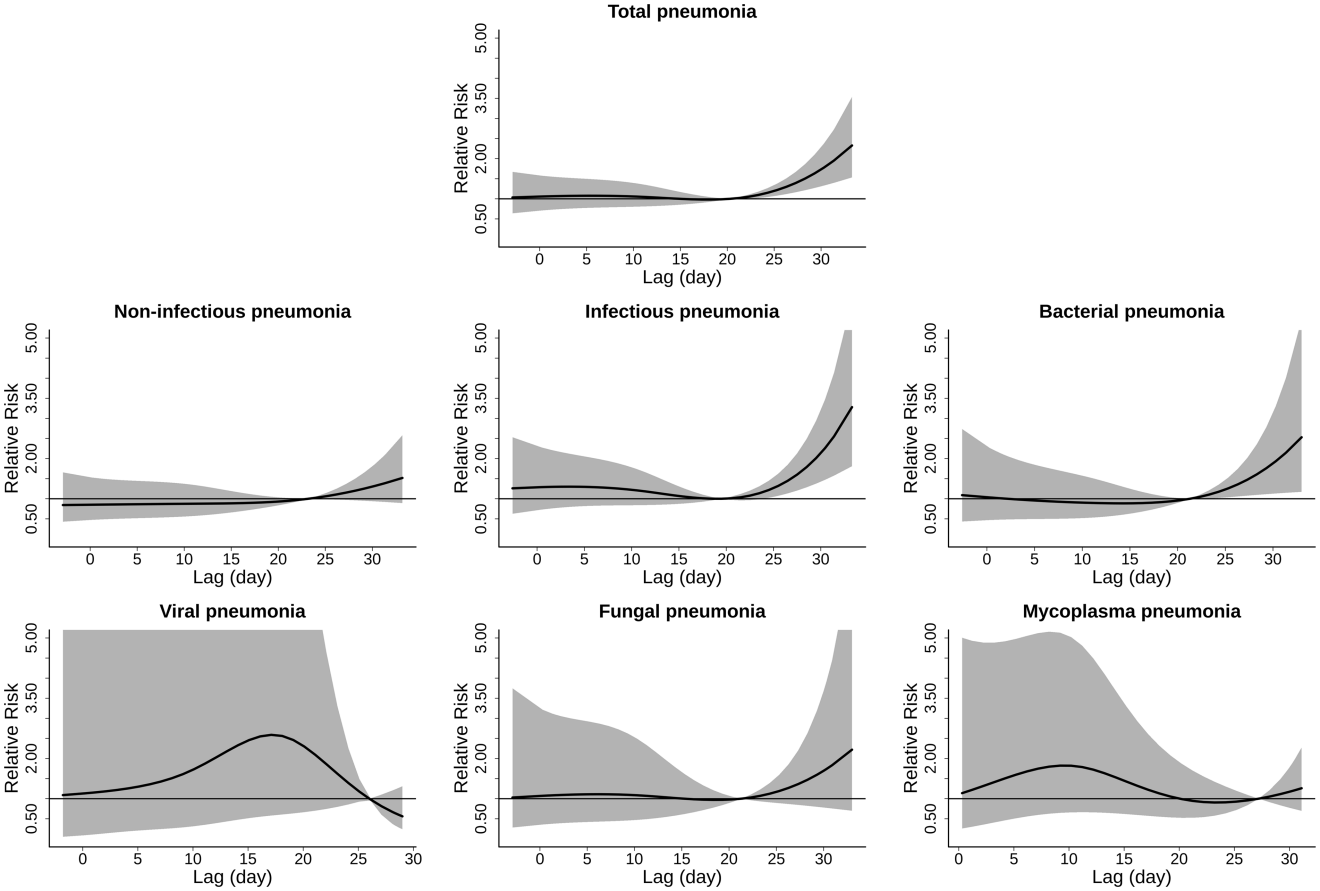
Cumulative exposure-response curves for the associations of ambient temperature with hospitalizations from cause-specific pneumonias. The associations were shown as relative risks and 95% confidence intervals of hospitalization comparing a given temperature to the corresponding reference temperatures. The mean risk estimates and their 95% confidence intervals are shown by the black solid lines and the gray areas.

The exposure-response curves of Figure 3 present the associations between extreme heat exposure and hospitalizations from cause-specific pneumonias. There were monotonically increasing trends of the risks with rising temperature, and the risks were statistically significant for extreme high temperature in total, infectious, and bacterial pneumonia. There were non-significant relationships in non-infectious, viral, fungal and mycoplasma pneumonia. Compared to the corresponding reference temperature, the RRs of hospitalizations from cause-specific pneumonias associated with extreme high temperature (99th percentile of temperature distribution) were 1.96 (1.40, 2.74) in total pneumonia, 2.55 (1.58, 4.13) in infectious pneumonia and 2.16 (1.15, 4.04) in bacterial pneumonia (Table 3). Infectious pneumonias exhibited the strongest sensitivity to extreme heat, followed by bacterial pneumonias and total pneumonias.

**Table 3:** Relative risks (95% CIs) of hospitalizations from cause-specific pneumonias associated with ambient low and high temperature.

Types	Reference temperature, °C	Extreme low temperature, °C	Relative risk	Extreme high temperature, °C	Relative risk
Total pneumonias	20.3	0.3	1.06 (0.71, 1.57)	31.4	1.96 (1.40, 2.74)
Non-infectious pneumonias	23.2	0.2	0.85 (0.47, 1.53)	31.4	1.39 (0.92, 2.11)
Infectious pneumonias	19.5	0.5	1.29 (0.73, 2.28)	31.4	2.55 (1.58, 4.13)
Bacterial pneumonias	21.3	0.3	1.03 (0.47, 2.26)	31.4	2.16 (1.15, 4.04)
Viral pneumonias	26.0	0.2	1.45 (0.11, 19.27)	31.6	0.91 (0.80, 1.03)
Fungal pneumonias	21.3	0.3	1.07 (0.36, 3.21)	30.9	1.83 (0.76, 4.44)
Mycoplasma pneumonia	26.8	0.8	1.22 (0.30, 4.93)	30.9	1.21 (0.74, 1.99)

As shown in Supplementary Table S2, we found that females and individuals aged above 60 years old were vulnerable to ambient high temperature, with significant associations in total, non-infectious, infectious, and bacterial pneumonia. Subgroup analyses showed that females had stronger risks, which indicated that we should take more attention and timely measures to protect vulnerable population from ambient high temperature.

Supplementary Table S3 displays that the associations of ambient high temperature and hospitalizations from cause-specific pneumonias were robust after adjusting for PM_2.5_, PM_2.5–10_, NO_2_, O_3_, CO, SO_2_, and all above pollutants, respectively. Additionally, our findings remained robust after adjusting for various model parameters of variables and DLNM model (Supplementary Tables S4–S8). Accounting for potential autocorrelation and collinearity, we found that all GVIF values of models were less than 4, which did not indicate collinearity among DLNM models.

## 4 Discussion

We provide new epidemiological evidence to the limited investigations about the differential impacts of non-optimum temperature on hospitalizations for cause-specific pneumonias, with an individual-level case-crossover design. These findings indicated that short-term exposure to ambient high temperature exerted adverse effects on hospitalizations from several types of pneumonias, including total, infectious, and bacterial pneumonia. Notably, infectious pneumonia had the highest risk estimate associated with exposure to extreme high temperature, followed by bacterial pneumonias. We did not observe significant associations between high temperature with other pneumonias, including non-infectious, viral, fungal and mycoplasma pneumonia. High risk estimates were observed in older individuals and females.

We observed nonlinear exposure-response curves for associations between extreme temperature and hospitalization from pneumonias, with increasing risks at high temperature, which was consistent with previous epidemiological studies (17, 33). It is widely believed that climatic factors could affect air-borne pathogens, thus affecting the prevalence of pneumonia (19, 21, 22). The multi-city time-series analyses in Texas and Madrid yielded that environmental heat exposure had a significant impact on pneumonia hospital admission with risk of 0.70% (95% CI, 0.35, 1.16%) (8, 34). A time-series study in Northern China reported that the risk of pneumonia hospitalization was 1.38 (95% CI, 1.24, 1.53) for extreme heat (35). Another time-series study in Jiuquan, China suggested that high temperature could increase the hospitalization risk of rural pneumonia, with significant effect at lag 0 day (RR = 1.008, 95% CI: 1.001, 1.020) (36). Our investigation had higher risk estimates, which may be attributed to different study design, analytical framework, and a few factors that are related to location. Moreover, our study utilized the exposure data derived from high-spatial-resolution models instead of the fixed-site monitoring data, which made our effect estimates more accurate. Although previous studies reported that ambient cold may increase the risk of pneumonia (12–15), we did not observe significant associations between ambient low temperature and various pneumonias. Consistently, a time-series investigation in Texas, America found that the association between low temperature and pneumonia was not statistically significant, with a RR of 7.0% (95% CI, −0.85, 15.46%) (16). The inconsistency existing in the cold-pneumonia association may be related to different characteristics of location, population, and behavioral factors (e.g., central heating and reduced outdoor activity).

Our study further revealed the differential associations of ambient high temperature with hospitalizations from various types of pneumonias. This study suggested that infectious pneumonia had higher risk estimate than non-infectious pneumonia, which aligned with previous investigations (22, 26). A case-crossover study in the Chinese mainland suggested that ambient heat exposure had significant association with the elevated risks of infectious pneumonia, and relative risks of influenza, viral and bacterial pneumonia were 1.49 (1.19, 1.86), 1.27 (1.16, 1.39) and 1.27 (1.19, 1.36), respectively (26). Generally, our findings suggested larger heat-related risks, which may be explained by the different study period, geographical location variations, and study population (i.e., higher proportion of older individuals in this study). In our study, infectious pneumonia had the highest heat-related risk estimate, which is aligned with previous studies (37, 38). Toxicological experiments on mice reported that exposure to high temperature could promote the infection of pneumococcal bacteria and the development of bacterial pneumonia (37). A multicenter case-crossover study in China also found that environmental high temperature could increase the incidence of hospitalized pneumonia (38). We did not observe the significant effects of extreme high temperature on viral, fungal and mycoplasma pneumonia hospitalization, which might relate to the relatively limited quantity of cases. Besides, high temperature is not conducive to virus transmission, which may explain the non-significant effect on viral pneumonia (39).

Ambient high temperature can elevate the risk of pneumonia by affecting physiological function and the physical environment. Extreme high temperatures may strain the core temperature regulation, disturb the heat balance, trigger physiological stress, cause cellular damage, enhance local inflammatory responses, and consequently increase the incidence risk of lung diseases (40, 41). Moreover, dehydration or heat stroke caused by heat stress may elevate bacterial autolysis, increase the release of key pneumococcal toxin pneumolysin, then alter the immune system function of the human body, and increase susceptibility to pneumonia infection (37, 42). In addition, extreme high temperature may rise the incidence risk of pneumonia by altering the physical environment. For instance, high temperatures are often accompanied by high humidity in Shanghai, which is conducive to the transmission of bacteria in some areas of the air (43). Moreover, the rapid increase in the release of ozone, particulate matter, and some organic pollutants at high temperature may elevate the risk of pneumonia through oxidative stress and systemic inflammation (8, 44).

Our study suggested that females had higher susceptibility to high temperature than males, which is in line with previous studies (23, 42). For instance, a national case-crossover study using the Urban Employee Basic Medical Insurance (UEBMI) database of 168 Chinese cities reported that heat exposure could increase hospital visits for pneumonia, and females have a greater vulnerability to environmental heat exposure (45). A multicenter time-series study in China also suggested that an increased risk of pneumonia was associated with heat exposure, and females were more susceptible to extreme high temperature (18). The sex differences in heat effect may be linked to the pathophysiological response to extreme temperature and socioeconomic status (30). Additionally, we observed non-significantly higher risks among the older population compared to the younger subgroup. Generally, the older adults may suffer from pre-existing diseases, which reduce the body’s ability to adapt to extreme high environments (40). Further studies are needed to explain the mechanisms of differential impacts of high temperature on pneumonia patients by gender and age.

Our findings have evidential policy significance and help to identify vulnerable subgroups in clinical management of pneumonia. We provide new evidence on environmental high temperatures and pneumonias of various causes, supporting the incorporation of heat exposure into pneumonia prevention and control. Our results suggest that it is necessary to take timely protective measures to reduce the heat-related burden of pneumonia. In the context of population aging, the incidence and mortality rates of pneumonia will continue to increase (3). Therefore, we should pay more attention to controlling risk factors for pneumonia and take measures to improve knowledge and protection against high temperature. For example, the government should provide public health support for high-risk groups, including building high-temperature warning systems, promoting the construction of air conditioning in public places, and enhancing public awareness and knowledge of self-protection among susceptible subgroups. In addition, taking effective protective measures at the individual level is of importance to reduce adverse health. For example, we can reduce outdoor activities in hot weather and use air conditioning to create a comfortable indoor environment.

Nevertheless, several limitations should be acknowledged. First, the medical records in analyses were derived from a single center, thus the results should be cautiously extrapolated to other regions with different climates and medical conditions. Second, exposure measurement errors are inevitable because individual-level exposure data were assessed based on residential address rather than personal monitoring devices, but it can be considered random and may cause lower observed impacts.

## 5 Conclusion

In conclusion, this individual-level, time-stratified, case-crossover study provides novel epidemiological evidence that short-term exposure to ambient high temperature could significantly increase the risks of hospitalizations from cause-specific pneumonias, including total, infectious, and bacterial pneumonia. Additionally, female pneumonia patients were more susceptible to high temperatures. Overall, our findings may provide valuable policy implications for the prevention and management of cause-specific pneumonias.

## Data Availability

The raw data supporting the conclusions of this article will be made available by the authors, without undue reservation.

## Footnotes

^1^ http://data.cma.cn/
