# Supplementary material for: Differentiating the impact of ambient temperature on hospitalization due to cause-specific pneumonias: an individual-level, case-crossover study
Source: Front Public Health. 2025 Oct 20;13:1615337. doi: 10.3389/fpubh.2025.1615337 (PMC12580377; doi:10.3389/fpubh.2025.1615337)
Supplement: Supplementary file 1 [file Data_Sheet_1.pdf]

Supplementary Material

Differentiating the impact of ambient temperature on hospitalization due to cause-specific pneumonias: An individual-level, case-crossover study

Table S1. Spearman correlation coefficients across air pollutants and meteorological conditions.

	$PM_{2.5}$	$PM_{2.5–10}$	NO_2	O_3	SO_2	CO	Temperature	Relative humidity	Precipitation	Wind speed
$PM_{2.5–10}$	0.54 *									
NO_2	0.68 *	0.46 *								
O_3	-0.03 *	0.06 *	-0.20 *							
SO_2	0.65 *	0.50 *	0.48 *	-0.08 *						
CO	0.61 *	0.34 *	0.44 *	-0.12 *	0.61 *					
Temperature	-0.44 *	-0.27 *	-0.54 *	0.53 *	-0.37 *	-0.23 *				
Relative humidity	-0.36 *	-0.34 *	-0.39 *	-0.23 *	-0.40 *	-0.19 *	0.37 *			
Precipitation	-0.20 *	-0.23 *	-0.20 *	-0.31 *	-0.23 *	-0.07 *	0.05 *	0.51 *		
Wind speed	-0.32 *	-0.11 *	-0.47 *	-0.11 *	-0.04 *	-0.11 *	-0.04 *	0.04 *	0.12 *	

Sunshine duration	0.06 *	0.18 *	0.01	0.57 *	0.18 *	0.01	0.20 *	-0.52 *	-0.60 *	-0.03 *

Abbreviations: $PM_{2.5}$, fine particulate matter; $PM_{2.5–10}$, coarse particulate matter; NO_2, nitrogen dioxide; O_3, ozone; SO_2, sulfur dioxide; CO, carbon monoxide. *: $P < 0.05$.

Table S2. Relative risks (95% CIs) of hospitalizations from cause-specific pneumonias associated with ambient high temperature, classified by sex, and age.

	Total pneumonia	Non-infectious pneumonia	Infectious pneumonia	Bacterial pneumonia	Viral pneumonia	Fungal pneumonia	Mycoplasma pneumonia
Total	1.96 (1.40, 2.74)	1.39 (0.92, 2.11)	2.55 (1.58, 4.13)	2.16 (1.15, 4.04)	0.91 (0.80, 1.03)	1.83 (0.76, 4.44)	1.21 (0.74, 1.99)
Sex							
Male	1.58 (0.95, 2.63)	0.96 (0.46, 2.01)	1.85 (0.97, 3.51)	2.26 (0.91, 5.62)	1.10 (0.14, 8.54)	2.07 (0.53, 7.98)	1.62 (0.42, 6.19)
Female	2.32 (1.43, 3.75)	2.93 (1.44, 6.00)	2.54 (1.26, 5.12)	3.60 (1.38, 9.37)	0.66 (0.12, 3.82)	2.55 (0.68, 9.63)	1.36 (0.44, 4.20)
P	0.28	0.03 *	0.51	0.49	0.72	0.83	0.85
Age							

<60	1.88 (1.20, 2.94)	1.58 (0.71, 3.53)	1.76 (0.96, 3.25)	1.60 (0.70, 3.54)	2.10 (0.49, 8.98)	1.61 (0.27, 9.45)	2.16 (0.70, 6.64)
≥60	2.06 (1.18, 3.59)	1.98 (1.02, 3.86)	2.67 (1.25, 5.71)	6.04 (2.04, 17.90)	0.41 (0.07, 2.45)	2.54 (0.90, 7.15)	0.85 (0.21, 3.37)
P	0.80	0.67	0.40	0.06	0.20	0.75	0.30

*: $P < 0.05$.

Table S3. Relative risks (95% CIs) of hospitalizations from cause-specific pneumonias associated with ambient high temperature, adjusting for air pollutants.

	Total pneumonia	Non-infectious pneumonia	Infectious pneumonia	Bacterial pneumonia	Viral pneumonia	Fungal pneumonia	Mycoplasma pneumonia
Total	1.96 (1.40, 2.74)	1.39 (0.92, 2.11)	2.55 (1.58, 4.13)	2.16 (1.15, 4.04)	0.91 (0.80, 1.03)	1.83 (0.76, 4.44)	1.21 (0.74, 1.99)
+$PM_{2.5}$	1.96 (1.40, 2.75)	1.41 (0.93, 2.14)	2.54 (1.57, 4.12)	2.19 (1.17, 4.11)	0.91 (0.81, 1.04)	1.85 (0.73, 4.66)	1.25 (0.76, 2.05)
+$PM_{2.5–10}$	1.97 (1.41, 2.75)	1.40 (0.93, 2.13)	2.56 (1.58, 4.15)	2.10 (1.15, 3.83)	0.92 (0.81, 1.04)	1.82 (0.75, 4.40)	1.06 (0.20, 5.70)
+NO_2	1.99 (1.42, 2.78)	1.38 (0.91, 2.10)	2.69 (1.63, 4.42)	2.12 (1.13, 3.98)	0.91 (0.80, 1.03)	1.96 (0.78, 4.94)	1.21 (0.74, 1.99)
+O_3	2.02 (1.44, 2.83)	1.48 (0.95, 2.32)	2.58 (1.61, 4.12)	2.20 (1.17, 4.14)	0.91 (0.81, 1.03)	1.99 (0.75, 5.26)	1.33 (0.70, 2.50)
+SO_2	1.96 (1.42, 2.70)	1.43 (0.95, 2.17)	2.55 (1.59, 4.07)	2.26 (1.20, 4.24)	0.92 (0.81, 1.04)	2.07 (0.79, 5.39)	1.09 (0.89, 1.34)
+CO	1.92 (1.39, 2.66)	1.43 (0.94, 2.17)	2.46 (1.53, 3.94)	2.23 (1.19, 4.19)	0.91 (0.80, 1.03)	2.14 (0.79, 5.80)	1.16 (0.81, 1.66)
+all pollutants	2.08 (1.48, 2.93)	1.54 (0.98, 2.41)	2.66 (1.65, 4.29)	2.15 (1.17, 3.94)	0.94 (0.83, 1.07)	2.62 (0.78, 8.78)	1.42 (0.74, 2.71)

Abbreviations as in Table S1.

Table S4. Relative risks (95% CIs) of hospitalizations from cause-specific pneumonias associated with ambient high temperature, adjusting for relative humidity with different lag periods.

	Total pneumonia	Non-infectious pneumonia	Infectious pneumonia	Bacterial pneumonia	Viral pneumonia	Fungal pneumonia	Mycoplasma pneumonia
Total	1.96 (1.40, 2.74)	1.39 (0.92, 2.11)	2.55 (1.58, 4.13)	2.16 (1.15, 4.04)	0.91 (0.80, 1.03)	1.83 (0.76, 4.44)	1.21 (0.74, 1.99)
over lag 0 to 7 days	2.00 (1.44, 2.77)	1.42 (0.97, 2.10)	2.55 (1.61, 4.03)	1.97 (1.11, 3.48)	0.98 (0.85, 1.12)	2.32 (0.80, 6.73)	1.02 (0.97, 1.08)
over lag 0 to 14 days	2.28 (1.55, 3.35)	2.28 (1.16, 4.50)	2.66 (1.61, 4.39)	2.35 (1.21, 4.57)	0.97 (0.85, 1.11)	3.56 (0.95, 13.39)	1.99 (0.31, 12.61)
over lag 0 to 21 days	2.25 (1.52, 3.34)	2.12 (1.10, 4.10)	2.51 (1.55, 4.05)	2.81 (1.27, 6.18)	0.95 (0.82, 1.09)	2.70 (0.75, 9.66)	1.03 (0.98, 1.08)

Abbreviations as in Table S1.

Table S5. Relative risks (95% CIs) of hospitalizations from cause-specific pneumonias associated with ambient high temperature, adjusting for relative humidity with different spline function.

	Total pneumonia	Non-infectious pneumonia	Infectious pneumonia	Bacterial pneumonia	Viral pneumonia	Fungal pneumonia	Mycoplasma pneumonia
Total	1.96 (1.40, 2.74)	1.39 (0.92, 2.11)	2.55 (1.58, 4.13)	2.16 (1.15, 4.04)	0.91 (0.80, 1.03)	1.83 (0.76, 4.44)	1.21 (0.74, 1.99)
B spline function	1.97 (1.41, 2.75)	1.40 (0.93, 2.12)	2.56 (1.58, 4.14)	2.17 (1.16, 4.07)	0.91 (0.80, 1.03)	1.86 (0.77, 4.51)	1.08 (0.88, 1.32)

Abbreviations as in Table S1.

Table S6. Relative risks (95% CIs) of hospitalizations from cause-specific pneumonias associated with ambient high temperature, adjusting for relative humidity with different degrees of freedom.

	Total pneumonia	Non-infectious pneumonia	Infectious pneumonia	Bacterial pneumonia	Viral pneumonia	Fungal pneumonia	Mycoplasma pneumonia
Total	1.96 (1.40, 2.74)	1.39 (0.92, 2.11)	2.55 (1.58, 4.13)	2.16 (1.15, 4.04)	0.91 (0.80, 1.03)	1.83 (0.76, 4.44)	1.21 (0.74, 1.99)
df = 4	1.96 (1.40, 2.75)	1.39 (0.92, 2.10)	2.57 (1.59, 4.17)	2.14 (1.14, 4.02)	0.91 (0.80, 1.03)	1.85 (0.76, 4.48)	1.22 (0.74, 2.01)
df = 5	1.95 (1.39, 2.72)	1.38 (0.91, 2.09)	2.56 (1.58, 4.16)	2.10 (1.12, 3.94)	0.91 (0.81, 1.04)	1.81 (0.75, 4.39)	1.31 (0.70, 2.45)
df = 6	1.95 (1.39, 2.72)	1.38 (0.91, 2.09)	2.56 (1.58, 4.15)	2.11 (1.13, 3.95)	0.91 (0.81, 1.04)	1.77 (0.73, 4.28)	1.30 (0.70, 2.44)

Abbreviations as in Table S1.

Table S7. Relative risks (95% CIs) of hospitalizations from cause-specific pneumonias associated with ambient high temperature, adjusting for different basic function of temperature in DLNM model.

	Total pneumonia	Non-infectious pneumonia	Infectious pneumonia	Bacterial pneumonia	Viral pneumonia	Fungal pneumonia	Mycoplasma pneumonia
Total	1.96 (1.40, 2.74)	1.39 (0.92, 2.11)	2.55 (1.58, 4.13)	2.16 (1.15, 4.04)	0.91 (0.80, 1.03)	1.83 (0.76, 4.44)	1.21 (0.74, 1.99)
B spline function	1.91 (1.37, 2.66)	1.36 (0.92, 2.01)	2.51 (1.50, 4.18)	2.22 (1.20, 4.13)	0.81 (0.64, 1.02)	1.80 (0.78, 4.11)	1.03 (0.16, 6.53)

Abbreviations as in Table S1.

Table S8. Relative risks (95% CIs) of hospitalizations from cause-specific pneumonias associated with ambient high temperature, adjusting for different *df* of temperature in DLNM model.

	Total pneumonia	Non-infectious pneumonia	Infectious pneumonia	Bacterial pneumonia	Viral pneumonia	Fungal pneumonia	Mycoplasma pneumonia
Total	1.96 (1.40, 2.74)	1.39 (0.92, 2.11)	2.55 (1.58, 4.13)	2.16 (1.15, 4.04)	0.91 (0.80, 1.03)	1.83 (0.76, 4.44)	1.21 (0.74, 1.99)
df = 4	1.86 (1.38, 2.51)	1.43 (0.91, 2.24)	2.47 (1.61, 3.79)	1.94 (1.11, 3.39)	1.01 (0.84, 1.20)	1.72 (0.61, 4.90)	1.15 (0.20, 6.71)
df = 5	1.97 (1.41, 2.74)	1.50 (0.93, 2.41)	2.54 (1.60, 4.03)	1.99 (1.13, 3.50)	0.93 (0.73, 1.18)	1.83 (0.40, 8.38)	0.85 (0.10, 7.26)
df = 6	1.82 (1.32, 2.52)	1.10 (0.98, 2.03)	2.44 (1.52, 3.89)	1.88 (1.06, 3.33)	0.76 (0.52, 1.11)	1.51 (0.32, 7.05)	0.83 (0.08, 8.96)

Abbreviations as in Table S1.
